# Prevalence of dental erosion in adolescent competitive swimmers exposed to gas-chlorinated swimming pool water

**DOI:** 10.1007/s00784-012-0720-6

**Published:** 2012-04-03

**Authors:** J. Buczkowska-Radlińska, R. Łagocka, W. Kaczmarek, M. Górski, A. Nowicka

**Affiliations:** Department of Conservative Dentistry, Pomeranian Medical University, Al. Powstańców Wlkp. 72, 70-111 Szczecin, Poland

**Keywords:** Competitive swimmers, Dental erosion, Erosion risk factors, Water saturation

## Abstract

**Objectives:**

The purpose of this study was to analyze the prevalence of dental erosion among competitive swimmers of the local swimming club in Szczecin, Poland, who train in closely monitored gas-chlorinated swimming pool water.

**Materials and methods:**

The population for this survey consisted of a group of junior competitive swimmers who had been training for an average of 7 years, a group of senior competitive swimmers who had been training for an average of 10 years, and a group of recreational swimmers. All subjects underwent a clinical dental examination and responded to a questionnaire regarding aspects of dental erosion. In pool water samples, the concentration of calcium, magnesium, phosphate, sodium, and potassium ions and pH were determined. The degree of hydroxyapatite saturation was also calculated.

**Results:**

Dental erosion was found in more than 26 % of the competitive swimmers and 10 % of the recreational swimmers. The lesions in competitive swimmers were on both the labial and palatal surfaces of the anterior teeth, whereas erosions in recreational swimmers developed exclusively on the palatal surfaces. Although the pH of the pool water was neutral, it was undersaturated with respect to hydroxyapatite.

**Conclusion:**

The factors that increase the risk of dental erosion include the duration of swimming and the amount of training. An increased risk of erosion may be related to undersaturation of pool water with hydroxyapatite components.

**Clinical relevance:**

To decrease the risk of erosion in competitive swimmers, the degree of dental hydroxyapatite saturation should be a controlled parameter in pool water.

## Introduction

Dental erosion is defined as the surface dissolution of dental hard tissues by acids without the involvement of microorganisms [1]. This process may be caused by a series of extrinsic and intrinsic factors. Extrinsic factors include the consumption of acidic foods and carbonated beverages, citrus fruits, low pH medications and, to a lesser degree, occupational exposure to acidic environments. Most clinical research has focussed on the impact of diet and lifestyle [1, 2]. Environmental acid exposure has also been associated with dental erosion and has been frequently documented in case studies [3]. Most prevalence studies relating to occupational dental erosion have been performed on workers at battery and galvanizing factories who are exposed to sulphuric acid and hydrochloric acid. However, several publications have indicated that competitive swimming may also constitute a risk factor for dental erosion. The first survey published by Savad [4] in 1982 revealed that swimmers in improperly maintained swimming pools may be susceptible to acid erosion of the enamel. Since that time, a few other reports have confirmed these findings [5, 6]. The epidemiological survey by Centerwall et al. [5] reported that 3 % of non-swimmers, 12 % of swimmers, and 39 % of swim team members suffer from dental erosion. Gabai et al. [6] analysed a hypothesis that dental erosion in competitive swimmers may be the result of low pH values in swimming pool water due to insufficient monitoring or inadequate buffering. A few case studies have shown rapid general dental erosion due to inadequate gas chlorination of swimming pool water. Geurtsen [7] described a case of a competitive swimmer with complete loss of enamel on the upper incisors and general erosion of the other teeth after a 27-day period of intensive swimming in improperly gas-chlorinated water. In 2008, Dawes and Boroditsky [8] described an almost complete loss of enamel by acid erosion in a woman who swam daily for 2 weeks in an improperly chlorinated swimming pool in Cuba. In contrast, Lokin and Huysmans [9] found that only 0.14 % of Dutch swimming pools have pH values below 5.5.

Public swimming pools are chlorinated to reduce bacterial and algal contamination. According to European Union (EU) regulations, the chlorine concentration in swimming pool water should be maintained within the range of 0.3–0.6 mg/dm^3^ [10]. Chlorine can be added to pool water as sodium hypochlorite, which has an alkaline pH and, thus, limited erosion potential. Such “stabilised chlorine” is created by combining chlorine and cyanuric acid salts. In large swimming pools, water is mainly chlorinated using chlorine gas [11]. Chlorine gas reacts with water to form hypochlorous acid (HOCl) and hydrochloric acid (HCl) in the reaction: Cl_2_ + H_2_O = HOCl + HCl. Hypochlorous acid is the germicidal agent in chlorination, whereas HCl is an unwanted by-product. The pH of the water is adjusted to approximately 7.5 by the addition of soda ash (Na_2_CO_3_). The accepted pH range for swimming pools is between 7.2 and 8.0 [12]. However, if a gas-chlorinated pool becomes inadequately buffered through the addition of insufficient quantities of soda ash, the pH may decrease rapidly to decalcifying levels as low as 3. Although swimmers may not sense the low pH, it may lead to tooth dissolution. The dental literature suggests that pool water with a low pH can cause very rapid and extensive dental erosion [5, 7, 8]. Therefore, intensive swimming should be considered a causative factor when diagnosing general dental erosion. These reports from the literature contribute to improving pool water disinfection and pH monitoring methods. First, according to American Public Health Association recommendations, a standard phenol red indicator system was used to check water pH values [12]. This system was also used in Poland, but the cases of tooth enamel loss described in the literature revealed deficiencies in the system. Currently, in most swimming pools in Szczecin, Poland, the water pH value is precisely monitored [10]. No studies were found in the literature regarding the effects of competitive swimming, in which several hours per day are spent in a properly maintained pool, on the susceptibility to dental erosion.

The purpose of this study was to analyse the prevalence of dental erosion among competitive swimmers from the local swimming club in Szczecin, Poland, who train in closely monitored gas-chlorinated swimming pool water.

## Subjects and methods

The population for this survey consisted of 14- to 16-year-old teenagers attending a swimming pool in Szczecin’s sport club. Participation was voluntary and required the parents’ approval. The Pomeranian Medical University Ethics Committee provided ethical approval for the study (No. KB-0012/42/11/10). The data were collected between October 2006 and January 2007.

The competitive swimmer study group consisted of 62 subjects (25 females, 37 males) who were competitors in the local sport clubs. The subgroup of junior competitive swimmers consisted of 24 subjects (12 females, 12 males) aged 14–15 years who had been training for an average of 7 years and spent over 19 h in the swimming pool per week. The subgroup of senior competitive swimmers consisted of 38 subjects (13 females, 25 males) aged 15–16 years who had been training for an average of 10 years and spent over 19 h in the swimming pool per week. The average duration of daily swimming sessions for both of these groups was 4 h. The recreational swimmer study group consisted of 69 subjects (34 females, 35 males) aged 14–16 years who were randomly selected from among students at Szczecin high schools who swam recreationally once or twice per week and spent no more than 2 h per week in the swimming pool. All subjects participating in the clinical study underwent a clinical dental examination using a standard dental mirror and dental explorer under artificial light. The examination included assessment of teeth condition with respect to dental erosion of the mineralised tooth tissue of all tooth groups. The lesions were assessed according to the Lussi Index [2]. The clinical examination was conducted by one oral pathologist who was blinded to whether the subjects were competitive swimmers.

Following the clinical examination, all study participants were asked to complete a structured questionnaire regarding aspects of their diet, medicine intake, tooth-brushing frequency, and swimming habits. For dietary acids, exposure was considered as regular consumption of acidic foods, such as citrus fruit and acidic beverages, twice or more per day. For acidic medicines, exposure was considered as regular intake of medicines such as effervescent vitamin C preparations, chewable vitamin C tablets, and iron tonics once or more per day. A question regarding “vomiting up of food” was used as a proxy for eating disorders.

The survey was conducted in the biggest swimming pool in Szczecin. The pool water was inspected and pool maintenance practices reviewed with the pool manager. The water was disinfected with chlorine gas and the routinely controlled parameter was chlorine concentration. The chlorine concentration was controlled every 3 h, according to EU regulations, and the measurements were recorded. In the present study, the records for the last 2 years were checked. During the course of this study, water samples were taken from two different corners and the middle of the pool every hour from 7 a.m. to 8 p.m., especially when the pool was fully occupied by swimmers over the course of 5 days. For all water samples, the pH value was determined using pH-indicator strips with a pH range of 6–10 and 0.3 accuracy (Merck KGaA, Germany). The concentrations of calcium, magnesium, phosphate, sodium, and potassium ions were also determined in all water samples using a DIONEX 3100 ion chromatograph. The degree of hydroxyapatite saturation of the pool water was determined using free software (Phreeqc, http://www.brr.cr.usgs.gov/projects/GWC_coupled/phreeqc/) provided by the U.S. Geological Survey (USGS).

Data were compared using Pearson’s chi-square test with Yates’ correction. All statistical analyses were performed using STATISTICA 6.0 software. A *p* value of 0.05 was considered significant.

## Results

The occurrence and localisation of dental erosion in competitive and recreational swimmers is provided in Table 1. Erosion was found in more than 26 % of competitive swimmers and 10 % of recreational swimmers (*p* = 0.02). Erosion occurred more frequently in males compared to females, but the difference was not significant. No differences were found in the stage of lesion development between the competitive and recreational swimmers or between genders. All lesions diagnosed as erosion, regardless of their location, developed in the surface enamel, rendering them Grade 1 lesions under the Lussi classification. No severe erosion with exposure of the dentin was diagnosed in any of the subjects. In competitive swimmers, erosion occurred on both the labial and palatal surfaces of the anterior teeth, whereas erosion developed exclusively on the palatal surfaces in recreational swimmers (Table 1). The lesions more frequently (*p* = 0.008) affected the labial surfaces of the central incisors in competitive swimmers compared to the palatal surfaces of the same teeth in recreational swimmers. All dental erosion observed in both competitive and recreational swimmers affected two or more teeth.

**Table 1:** Occurrence and localisation of dental erosions in competitive and recreational swimmers

		Competitive swimmers (*n* = 62)	Recreational swimmers (*n* = 69)
Subjects with two or more affected teeth	Total *n* (%)	16 (26 %)	7 (10 %)
	Male	12 (19 %)	5 (7 %)
	Female	4 (6 %)	2 (3 %)
Subjects with affected labial surface	Total n (%)	6 (10 %)	0 (0 %)
	Male	5 (8 %)	0 (0 %)
	Female	1 (2 %)	0 (0 %)
Subjects with affected palatal surface	Total n (%)	10 (16 %)	7 (10 %)
	Male	7 (11 %)	5 (7 %)
	Female	3 (5 %)	2 (3 %)

The occurrence and localisation of dental erosion in junior and senior competitive swimmers is provided in Table 2. Erosion was more prevalent in senior competitive swimmers than junior competitive swimmers, but the difference was not significant. Dental erosion on palatal tooth surfaces was observed in both junior and senior competitive swimmers and with similar frequency. Affected labial tooth surfaces were detected in only senior competitive swimmers (*p* = 0.04), more frequently in males compared to females (*p* > 0.05).

**Table 2:** Occurrence and localisation of dental erosions in junior and senior competitive swimmers

		Junior competitive swimmers (*n* = 38)	Senior competitive swimmers (*n* = 24)
Subjects with two or more affected teeth	Total, *n* (%)	4 (11 %)	12 (50 %)
	Male	4 (11 %)	10 (42 %)
	Female	0 (0 %)	2 (8 %)
Subjects with affected labial surface	Total, *n* (%)	0 (0 %)	6 (25 %)
	Male	0 (0 %)	5 (21 %)
	Female	0 (0 %)	1 (4 %)
Subjects with affected palatal surface	Total, *n* (%)	4 (11 %)	6 (25 %)
	Male	4 (11 %)	4 (17 %)
	Female	0 (0 %)	2 (8 %)

Diseases associated with vomiting and other eating disorders that lower pH in the oral cavity were not detected in any subject. Both competitive (45 %) and recreational (74 %) swimmers frequently consumed dietary acids (Table 3). Recreational swimmers more frequently (*p* = 0.001) consumed dietary acids compared to competitive swimmers, and senior competitive swimmers more frequently (*p* = 0.001) consumed dietary acids compared to junior competitive swimmers. No difference was found between genders in regards to dietary acid consumption. Also, no significant difference was found in the consumption of acidic medicines between competitive and recreational swimmers or between genders. More competitive swimmers reported brushing their teeth more than twice a day compared to recreational swimmers (40 % vs. 27 %), but this difference was not significant.

**Table 3:** Competitive and recreational swimmers exposed to erosion risk factors

Erosion risk factor		Junior competitive swimmers (*n* = 38)	Senior competitive swimmers (*n* = 24)	Recreational swimmers (*n* = 69)
Dietary acids	Total, *n* (%)	8 (21 %)	20 (83 %)	51 (74 %)
	Male	4 (11 %)	14 (58 %)	25 (36 %)
	Female	4 (11 %)	6 (25 %)	26 (38 %)
Acidic medicines	Total, *n* (%)	3 (8 %)	7 (29 %)	11 (16 %)
	Male	0 (0 %)	3 (12 %)	7 (10 %)
	Female	3 (8 %)	4 (17 %)	4 (6 %)

Analysis of the data obtained from the pool manager found that the chlorine concentration of the pool water was maintained within the range of 0.3–0.5 mg/dm^3^ over the last 2 years. The pH of the water fluctuated between 6.8 and 8.0, with a median pH of 7.2. These differences did not depend on time of day or the sampling location. The mean concentrations of calcium, phosphate, sodium, potassium, magnesium, and chlorate ions in the pool water and saturation with respect to hydroxyapatite are presented in Table 4. The data show that the pool water with a pH of 7.2 was undersaturated with respect to hydroxyapatite.

**Table 4:** Chemical concentrations measured in pH 7.2 pool water and saturation indices with respect to hydroxyapatite

Calcium	Phosphate	Sodium	Potassium	Magnesium	Chlorate
2.2 ± 0.03	0.0003 ± 0.00001	5.4 ± 0.03	0.2 ± 0.01	0.8 ± 0.04	0.2 ± 0.06
Saturation index	Log of the ion activity product			Log of the solubility constant	
−3.33	−6.75			−3.42	

Concentrations are given as mean ± SD, mmol/l (*n* = 5)

## Discussion

Lesions in competitive swimmers caused by a low pH of the pool water are mainly described as general erosion [5, 7, 8], and the most affected tooth surfaces are the labial surfaces of the upper incisors. In the present study, no general erosion was noted and every observed erosion lesion was classified as Grade 1 according to the Lussi Index. These lesions correspond to Grade 1 of a new scoring system criteria for grading erosive wear of teeth — Basic Erosive Wear Examination (BEWE) [13]. This classification implements simpler criteria for grading erosive wear than the Lussi Index and it seems to be simple, fast and standardised tool suitable for monitoring erosion activities such as progression or arrest of lesions in competitive swimmers group.

The observed lesions more frequently affected the labial surfaces of the mesial maxilla incisors in senior competitive swimmers. Thus, the labial surfaces of maxilla incisors seem to be subject to more erosion factors related to competitive swimming in our study, which may be due to these teeth having continued contact with pool water and no protective actions of saliva [14]. All diagnosed erosion developed in the surface enamel and no severe erosion with exposure of the dentin or dentin hypersensitivity was diagnosed in any of the study subjects. Therefore, the erosive factor was sporadically active or its damaging potential was low. In earlier studies describing cases of enamel erosion in people using swimming pools with poorly controlled disinfectant dosing, the main cause of tooth defects was the low pH of the pool water [5–9]. Low pH is not exclusively responsible for the dental hydroxyapatite dissolution process, but the concentration of ions with respect to the saturation of dental hydroxyapatite, especially calcium and phosphate, may also be involved. Therefore, even in water with a neutral pH value, dental hydroxyapatite dissolution processes can occur if the pool water is undersaturated. A small degree of undersaturation with respect to enamel or dentine leads to initial surface demineralization, followed by a local increase in pH and increased mineral content in the liquid surface layer adjacent to the tooth surface. This layer will then become saturated with respect to dental tissue and will not demineralise further [15]. In the present study work, the pool water, despite having a pH above 7.0, was undersaturated with respect to hydroxyapatite. Long-term exposure to such pool water was probably the main factor responsible for damage to the labial teeth surfaces in senior competitive swimmers. Labial erosions were found more frequently in males, which was related to the longer sessions and more aggressive style of men’s swimming, involving increased agitation (e.g., when a swimmer is swishing water in the mouth). This activity enhances the dissolution process because the solution on the surface layer adjacent to the enamel is readily renewed. In addition, the amount of water in the mouth in relation to the amount of saliva modifies the dissolution process [16]. Both competitive and recreational swimmers with dental erosion consumed dietary acids. Dietary acid consumption is regarded as the most common reason for tooth lesions and was probably responsible for erosion development on palatal tooth surfaces in both competitive and recreational swimmers [15, 16].

## Conclusion

Factors that increase the risk of dental erosion include the duration of swimming and the amount of training. The risk of erosion on the labial surface of the central incisors in competitive swimmers was greater than in recreational swimmers. The increased risk of erosion may be related to the undersaturation of pool water with respect to hydroxyapatite components. Thus, the degree of dental hydroxyapatite saturation should be a controlled parameter in pool water to decrease the risk of erosion in competitive swimmers.
